# Tankyrases maintain homeostasis of intestinal epithelium by preventing cell death

**DOI:** 10.1371/journal.pgen.1007697

**Published:** 2018-09-27

**Authors:** Pan Ye, Y. Jeffrey Chiang, Zhen Qi, Yehua Li, Shan Wang, Yuan Liu, Xintong Li, Ye-Guang Chen

**Affiliations:** 1 The State Key Laboratory of Membrane Biology, Tsinghua-Peking Center for Life Sciences, School of Life Sciences, Tsinghua University, Beijing, China; 2 Experimental Immunology Branch, NCI, National Institutes of Health, Bethesda, Maryland, United States of America; Harvard University, UNITED STATES

## Abstract

Lgr5^+^ intestinal stem cells are crucial for fast homeostatic renewal of intestinal epithelium and Wnt/β-catenin signaling plays an essential role in this process by sustaining stem cell self-renewal. The poly(ADP-ribose) polymerases tankyrases (TNKSs) mediate protein poly-ADP-ribosylation and are involved in multiple cellular processes such as Wnt signaling regulation, mitotic progression and telomere maintenance. However, little is known about the physiological function of TNKSs in epithelium homeostasis regulation. Here, using *Villin-cre*ERT2;*Tnks1*^-/-^;*Tnks2*^fl/fl^ (DKO) mice, we observed that loss of TNKSs causes a rapid decrease of Lgr5^+^ intestinal stem cells and magnified apoptosis in small intestinal crypts, leading to intestine degeneration and increased mouse mortality. Consistently, deletion of *Tnks* or blockage of TNKS activity with the inhibitor XAV939 significantly inhibits the growth of intestinal organoids. We further showed that the Wnt signaling agonist CHIR99021 sustains the growth of DKO organoids, and XAV939 does not cause growth retardation of *Apc*^*-/-*^ organoids. Consistent with the promoting function of TNKSs in Wnt signaling, Wnt/β-catenin signaling is significantly decreased with stabilized Axin in DKO crypts. Together, our findings unravel the essential role of TNKSs-mediated protein parsylation in small intestinal homeostasis by modulating Wnt/β-catenin signaling.

## Author summary

Although tankyrases have been indicated to play important roles in telomere maintenance, mitosis and Wnt signaling regulation, little is known about their physiological function in intestinal epithelium. Using *Villin-cre*ERT2;*Tnks1*^-/-^;*Tnks2*^fl/fl^ mice, which harbored conventional *Tnks1* deletion and inducible intestinal epithelium-specific *Tnks2* knockout, we show that tankyrases regulate adult intestinal *Lgr5*+ stem cells and epithelium homeostasis by preventing cell death and promoting cell proliferation.

## Introduction

The intestinal epithelium carries out many physiological functions including absorbing nutrients and providing barrier function [1, 2]. The intestinal epithelium is one of the most rapidly renewing tissues in adult mammals with renewal time of about every 5 days [3, 4]. This continual renewal is maintained by actively proliferating Lgr5^+^ intestinal stem cells (ISCs) located at the base of intestinal crypts [1, 5, 6]. Several signaling pathways control intestinal epithelial homeostasis by regulating cell proliferation, differentiation and cell death, such as Wnt, Notch, Hippo and bone morphogenetic protein (BMP) signaling pathways [7–10]. It has been well documented that Wnt signaling is of vital importance in the maintenance of ISCs [11–13]. Consistently, most of the intestinal stem cell enriched genes, referred to as stem cell signature genes, are the target genes of Wnt signaling [4, 14]. Wnt ligands evoke the signaling process via binding to their transmemebrane receptors Frizzled and low-density lipoprotein receptor-related protein (LRP)-5/6, which then recruit Dishevelled and Axin, respectively. These events cause the disassembly of the β-catenin destruction complex consisting of Dishevelled, Axin, adenomatous polyposis coli (APC), glycogen synthase kinase (GSK)-3, casein kinase and others, leading to accumulation of β-catenin that activates transcription of Wnt target genes [15, 16]. As a negative regulator of Wnt/β-catenin signaling, Axin degradation can promote Wnt signaling [17, 18].

Tankyrases (TNKSs) belong to the poly(ADP-ribose) polymerase (PARP) superfamily, consisting of function-redundant TNKS1 and TNKS2 in vertebrates [19]. TNKSs catalyze poly(ADP-ribosylation) or parsylation by generating the long chain ADP-ribose polymer in their target proteins, and this modification regulates the stability of the proteins involved in many processes [20–22]. TNKSs regulate Wnt/β-catenin signaling [23], mitosis [24, 25], telomere length [26, 27] and other processes [28, 29]. TNKSs can interact with Axin, mediate its parsylation and then stimulate its degradation through the ubiquitin-proteasome pathway, therefore promoting Wnt/β-catenin signaling [23].

TNKSs are widely expressed in many human organs, including small intestine, colon and placenta [30, 31]. *Tnks1/2* double knockout leads to embryonic lethality in mice [19], while *Tnks2* knockout mice exhibit mild phenotypes, such as decreased body weight, especially in male mice [32, 33], indicating that these two genes have redundant functions. Therefore, the physiological functions of TNKSs in adult tissue homeostasis of mammals are still poorly understood. Recently, it has been shown that deletion of the *Drosophila* TNKS homolog caused no obvious macroscopic phenotypes under normal feeding condition, but increased ISC number surrounded by *Tnks*-deficient enterocyte [34].

To better understand the functions of TNKSs in intestinal physiology, we generated *Villin-cre*ERT2;*Tnks1*^-/-^;*Tnks2*^fl/fl^ (DKO) mice. Our results revealed that TNKS DKO led to defective crypts with significant loss of both *Lgr5*^+^ ISCs and *Ki67*^+^ transient amplifying cells, resulting in an obvious increase of mortality in long term. Consistent with the promoting role of TNKSs in Wnt signaling, Axin level was increased while Wnt signaling activity was decreased in TNKS DKO intestinal epithelial cells. These data were confirmed with the TNKS inhibitor XAV939 in *in vitro* cultured organoids. Taken together, our findings indicate that TNKSs play an essential role in intestinal epithelium homeostasis by modulating Wnt/β-catenin signaling.

## Results

### Double knockout of *Tnks1* and *Tnks2* causes lethal degeneration of intestinal crypts

It has been shown that TNKS1 and TNKS2 are highly expressed in both small intestine and colon [30, 31]. We confirmed that both TNKS1 and TNKS2 were expressed in the intestinal epithelium (S1A Fig). Importantly, immunohistochemical analysis showed that TNKSs exhibited a gradually decreased expression from the crypt bottom to the villus tip in small intestine (S1A Fig). This expression pattern is consistent with the Wnt signaling activity gradient along the crypt-villus axis [7, 8].

**Fig 1:**
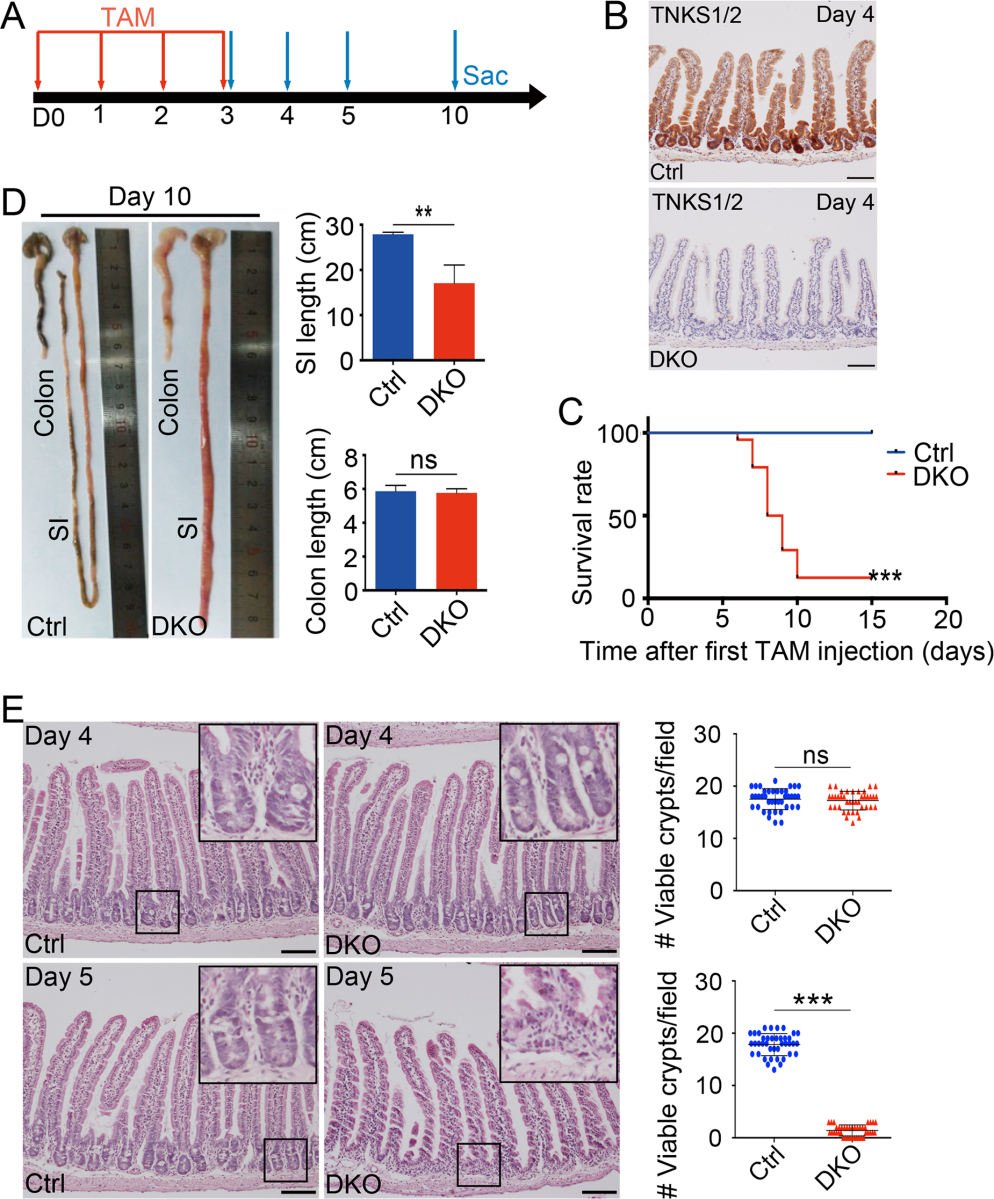
*Tnks* DKO results in intestine degeneration and mouse lethality. (A) Experimental outline for intestine-specific deletion of *Tnks2*. TAM: tamoxifen; Sac: sacrifice. (B) Immunohistological analysis of TNKSs knockout efficiency in small intestinal epithelium at day 4 after the first TAM injection. Representative images from three mice of each genotype are depicted. Scale bar: 100 μm. Ctrl and DKO indicate *Tnks1*^+/+^;*Tnks2*^+/+^ and *Vil-creERT2*;*Tnks1*^-/-^;*Tnks2*^fl/fl^, respectively. (C) Survival rate of control and DKO (n = 24) mice, administered TAM intraperitoneally daily for 4 times, from two different experiments. ***P < 0.001, by log rank test. (D) Left panel: Representative images of small intestines and colons from control and DKO mice at day 10 after the first TAM administration. Right panel: The statistic results of the small intestine and colon length (n = 5 mice). (E) H&E staining of small intestinal sections from the indicated adult mice at day 4 or day 5 after the first TAM administration. Left panel: Representative images from 5 mice of each genotype are depicted. Scale bar: 100 μm. Right panel: The statistics of the number of viable crypts of the indicated adult mice (n = 4). Data are represented as means ± SD, analyzed by two-way ANOVA test. *P < 0.05, **P < 0.01, ***P < 0.001.

Conventional *Tnks* knockout in mice showed that TNKS1 and TNKS2 are functionally redundant, and *Tnks* double knockout resulted in embryonic lethality [19]. In order to investigate the role of TNKSs in the maintenance of intestinal epithelium homeostasis, we generated *Villin-cre*ERT2;*Tnks1*^-/-^;*Tnks2*^fl/fl^ (DKO) mice, which harbored conventional *Tnks1* deletion and inducible intestinal epithelium specific *Tnks2* knockout. At 8–10 weeks of age, *Villin-cre*ERT2;*Tnks1*^-/-^;*Tnks2*^fl/fl^ (DKO) mice and control (Ctrl) littermates (*Tnks1*^-/-^;*Tnks2*^fl/fl^) were daily injected with tamoxifen (TAM) for 4 consecutive days to induce *Tnks2* deletion, then sacrificed at different time points after their first injection (Fig 1A). By immunoblotting and immunohistochemical staining, we detected a near-complete loss of both TNKS1 and TNKS2 in DKO intestines at 4 day after the first TAM injection (Fig 1B; S1B and S1C Fig). Interestingly, DKO mice showed a rapid onset of weight loss from day 6 (S1D Fig). By day 10, DKO mice displayed diarrhea and small intestine bleeding (S1E and S1F Fig), and showed remarkably increased mortality (Fig 1C). Overall, the small intestines from these DKO mice were shortened and appeared distended and necrotic (Fig 1D and S1F Fig). However, colons appeared normal.

In line with the mortality and gross abnormality phenotypes in DKO mice, although little changes were detected at day 4, severely degenerative crypts appeared in small intestine at day 5, and shortened and irregular villi were observed at day 10 (Fig 1E and S1G Fig). Most crypts were disorganized and the stem cell zone consisting of Paneth cells and intestinal stem cells (ISCs) disappeared. At day 10, we could also detect some hyperplastic crypts, which might be the escaping crypts as such escaping crypts are frequently observed upon essential gene ablation [35, 36]. Although a great amount of degenerative crypts with few escaping crypts were present throughout small intestine, but colon just showed a few degenerative crypts with mildly decreased viable crypts in day 10 DKO mice (S1H Fig). Together, *Tnks* deficiency caused the lethal degeneration of small intestinal crypts, indicating that, TNKSs are essential for small intestinal homeostatic self-renewal.

### Loss of TNKSs reduces proliferation and induces apoptosis in small intestinal crypts

**Fig 2:**
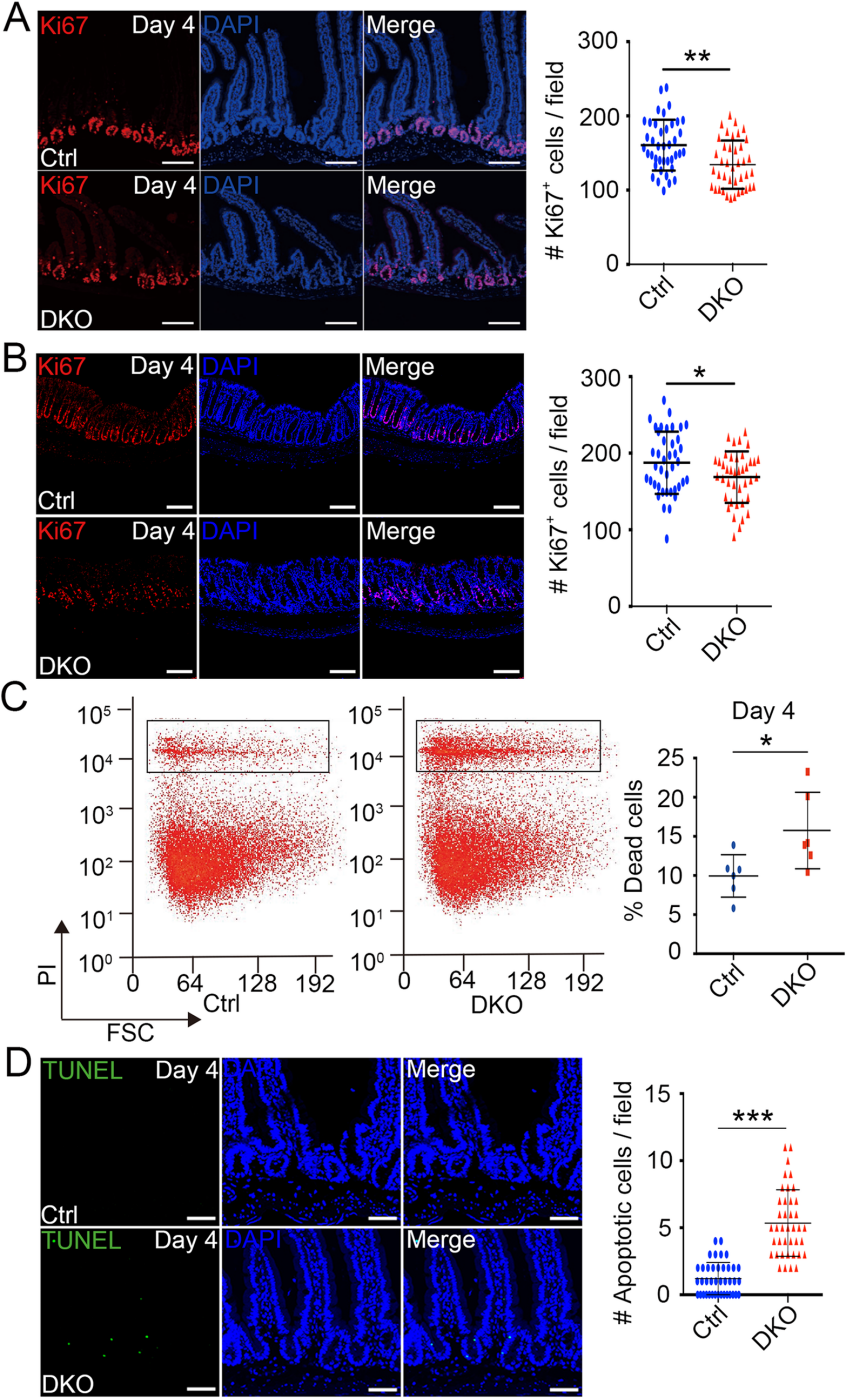
Reduced cell proliferation and increased apoptosis appear in *Tnks* DKO crypts. (A, B) Immunofluorescence analysis of small intestinal (A) or colon (B) sections from mice at day 4 after the first TAM injection shows the expression of the proliferation marker Ki67. Left panel: Representative images from five mice of each genotype are depicted. Scale bar: 80 μm (A) and 100 μm (B). Right panel: Quantification of the Ki67 positive cells in crypts (n = 4 mice). (C) Small intestine crypt cells were isolated from control and *Tnks* DKO mice at day 4 after the first TAM injection, stained by propidium iodide (PI), and then subjected to FACS analysis. Left panel: A representative FACS result from 5 mice of each genotype is depicted. Right panel: Quantification of the percentage of dead cells (n = 6 mice). (D) TUNEL assay of small intestine of mice at day 4 after the first TAM injection. Left panel: Representative images from 4 mice of each genotype are depicted. Cell nuclei were counterstained with DAPI. Scale bar: 50 μm. Right panel: Quantification of apoptotic cells of the indicated adult mice (n = 4). 40 fields were scored for each condition. Data are represented as means ± SD, analyzed by two-way ANOVA test. *P < 0.05, **P < 0.01, ***P < 0.001.

We then examined cell proliferation in intestine. At day 2, Ki67 staining indicated that cell proliferation in crypts was normal (S2A Fig). At day 3 and 4 DKO mice, although the number of viable crypts had no obvious change, the proliferative cells in crypts marked by Ki67 were decreased in small intestine (Fig 2A and S2B Fig). At day 5, the majority of proliferating crypts disappeared, and only few crypts had limited proliferating cells (S2C Fig). Similarly, proliferating cells in colon were also notably reduced in day 4 DKO mice (Fig 2B), and few proliferative cells were remained at day 5 (S2D Fig).

To further explore the degenerative crypt phenotype, we examined cell death in crypts of small intestine by FACS basing on propidium iodide (PI) staining. The result showed that *Tnks* deletion led to about 50% increase of cell death at day 4 mice, which was consistent with the TUNEL assay results (Fig 2C and 2D; S3A–S3C Fig). Similarly, more cell death was observed in DKO colon (S3D Fig). These data together suggest that the degenerative crypt phenotype in DKO mice is due to both reduced proliferation and enhanced apoptosis.

### *Tnks* deficiency causes loss of *Lgr5*^+^ intestinal stem cells

**Fig 3:**
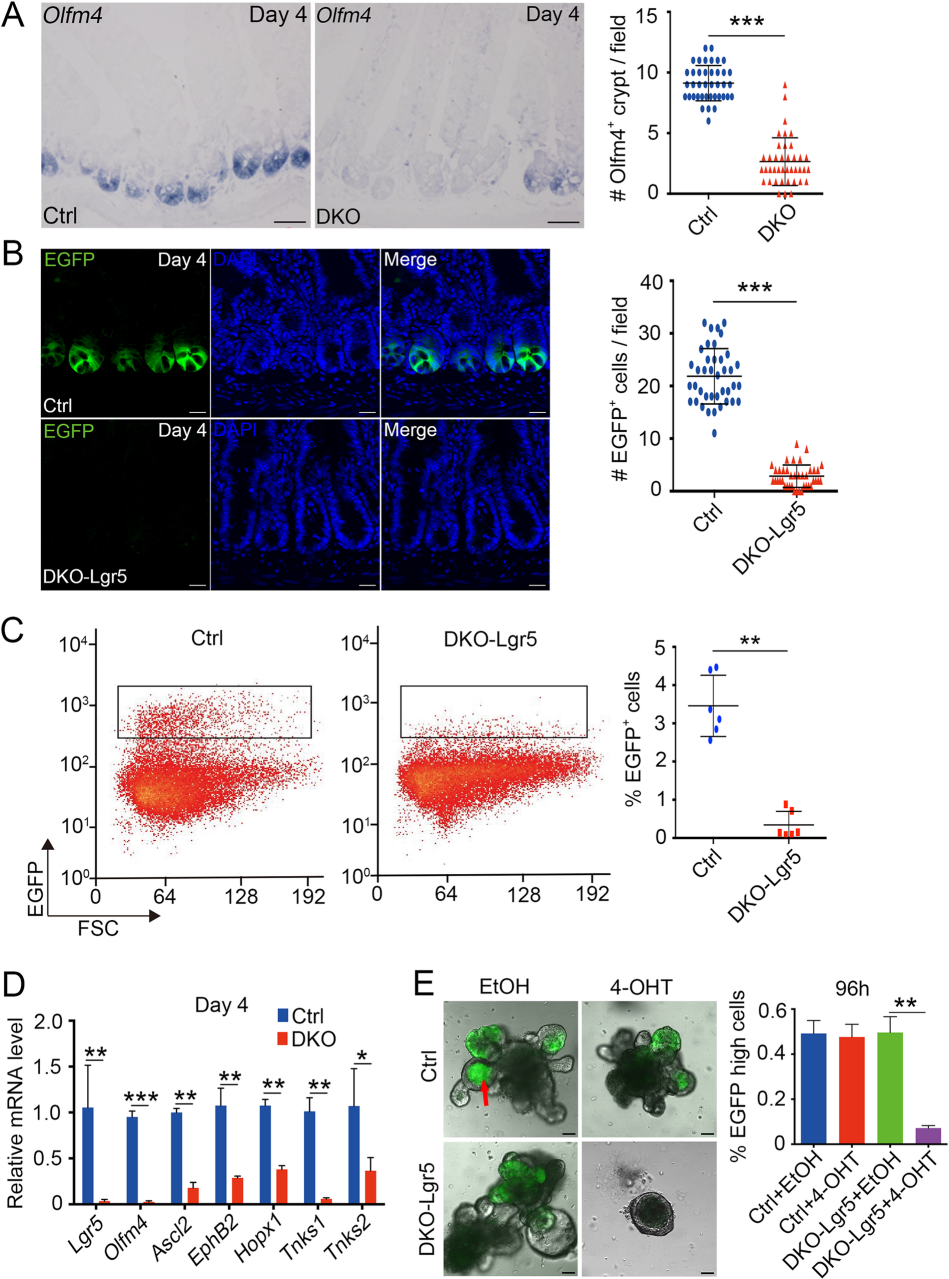
Ablation of TNKSs reduces intestinal Lgr5^+^ stem cells. (A) *In situ* hybridization analysis of intestinal stem cell marker *Olfm4* expression in small intestine sections of control and *Tnks* DKO mice at day 4 after the first TAM injection. Left panel: Representative images from 4 mice of each genotype are depicted. Scale bar: 50 μm. Right panel: Quantification of the *Olfm4* positive crypts of the indicated adult mice (n = 4). 40 fields were scored for each condition. (B) Lgr5-EGFP-expressing cells in proximal jejunum sections of Ctrl (*Lgr5-EGFP-IRES-creERT2*) or DKO-Lgr5 (*Lgr5-EGFP-IRES-creERT2*;*Tnks1*^-/-^;*Tnks2*^fl/fl^) mice at day 4 after the first TAM injection. Cell nuclei were counterstained with DAPI. Left panel: Representative images from 4 mice of each genotype are depicted. Scale bar: 50 μm. Right panel: Quantification of the GFP-positive cells of the indicated adult mice (n = 4). 40 fields were scored for each condition. (C) FACS analysis of Lgr5-EGFP-positive ISCs from small intestine crypt of the mice at day 4 after first TAM injection. Right panel: Quantification of EGFP^+^ cells of the indicated adult mice (n = 6). (D) Crypts of indicated mice (n = 3) at day 4 after the first TAM injection were isolated for qRT-PCR analysis of intestinal stem cell marker gene expression. (E) Organoids derived from the crypts of mice in (B) were treated by vehicle (EtOH) or 4-OHT. Left panel: Representative images were taken at 96h, from 5 independent experiments. Scale bar: 100 μm. Notes: Red arrow indicates autofluorescence in the organoid lumen. Right panel: Quantification of Lgr5-EGFP-high cells. Data from 5 independent experiments are represented as mean ± SEM, analyzed by unpaired Student’s t-test. **P<0.01. Data (A, B, C and D) are represented as means ± SD, analyzed by two-way ANOVA test. *P < 0.05, **P < 0.01, ***P < 0.001.

We suspected that crypt degeneration is caused by loss of ISCs that fueling the self-renewal of crypts [6, 37]. First, we performed *in situ* hybridization of *Olfm4*, a stem cell marker for *Lgr5*^+^ ISCs [14, 38]. As shown in Figs 3A, S4A and S4B, *Tnks* DKO intestine showed dramatically decreased *Olfm4* expression as early as at day 2. In order to quantify the number of ISCs, we generated *Lgr5-EGFP-IRES-creERT2*;*Tnks1*^-/-^;*Tnks2*^fl/fl^ (DKO-Lgr5) mice, in which green fluorescent protein could mark *Lgr5*-positive ISCs. A marked decrease of GFP^+^ cells was observed in day 4 *Tnks* DKO mice (Fig 3B), which was confirmed by FACS analysis (Fig 3C). We further employed quantitative reverse-transcription PCR (qRT-PCR) to assess the expression of the ISC markers and detected the down-regulation of *Lgr5*, *Olfm4*, *Ascl2*, *EphB2* and *Hopx1* mRNAs in *Tnks* deficient crypts as early as at day 2 (Fig 3D and S3C Fig). Furthermore, loss of Lgr5^+^ ISCs in cultured organoids was also observed upon 4-hydroxytamoxifen (4-OHT)-induced *Tnks* DKO (Fig 3E). These data suggest that TNKSs are indispensable for the Lgr5^+^ ISC maintenance in adult small intestine.

### *Tnks* deficiency reduces Paneth cells and goblet cells

**Fig 4:**
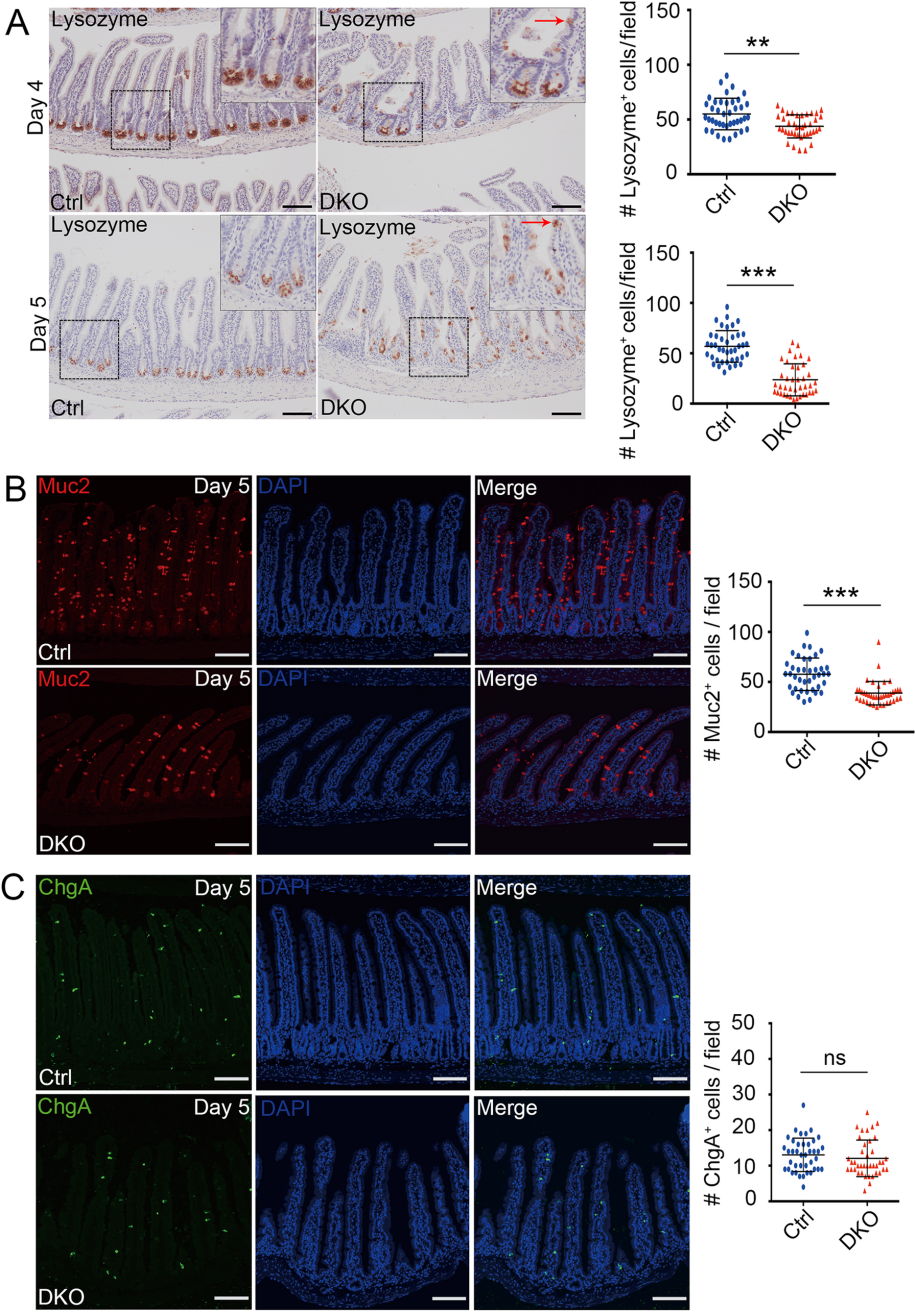
Decrease of Paneth cells and goblet cells in small intestine upon deletion of TNKSs. (A) Histological analysis of Paneth cells by lysozyme staining in mice at day 4 and 5 after the first TAM injection. Representative images from four mice of each genotype are depicted. Notes: Red arrow indicates dislocated Paneth cells. Scale bar: 100 μm. Right panel: Quantification of Lysozyme^+^ cells (n = 4 mice). (B, C) Mucin 2 (B) or Chromogranin A (C) immunofluorescence staining of small intestinal sections from mice on day 5 after the first TAM injection. Cell nuclei were counterstained with DAPI. Scale bar: 100 μm. Right panel: Quantification of goblet cells (B) or enteroendocrine cells (C) from 40 fields scored for each condition (n = 4 mice). Data are represented as means ± SD, analyzed by two-way ANOVA test. **P < 0.01 and ***P < 0.001.

As Paneth cells are a crucial component of the Lgr5^+^ ISC niche [39], we explored whether *Tnks* knockout affects Paneth cells by lysozyme immunohistochemical staining. As shown in Fig 4A, at day 4 and day 5 after first TAM injection, Paneth cells in crypt bottom were significantly diminished, and some of them were found to be mislocated in villi. Muc2 immunofluorescence staining showed that the goblet cell number was also significantly reduced in day 5 DKO mice (Fig 4B), while the number of enteroendocrine cells (marked by chromogranin A), remained unchanged in control and DKO mice (Fig 4C). Together, these data indicated that TNKSs play a critical role in the maintenance or differentiation of Paneth cells and goblet cells.

### Ablation of TNKSs impairs Wnt/β-catenin signaling in intestinal epithelial cells

TNKSs play an important role in regulating Wnt/β-catenin signaling and are involved in many cellular processes, including mitosis, telomere maintenance and others. As cell proliferation was greatly reduced in DKO intestine and TNKS1 knockdown led to the formation of multipolar mitotic spindle or mitotic arrest, thus inhibiting the cell division [24, 40], we analyzed the mitotic spindle morphology. Tubulin staining indicated that the bipolar mitotic spindle were normal in day 4 DKO intestinal crypt cells (S5A Fig). Although it was shown that the cell cycle arrest can lead to the formation of multinucleated cells shown by nuclear lamina protein Lamin B staining [41], we found no obvious multinucleated cells in small intestinal crypts of day 4 and day 5 DKO mice (S5B Fig). Together, these data suggested that mitosis was normal in DKO intestine.

**Fig 5:**
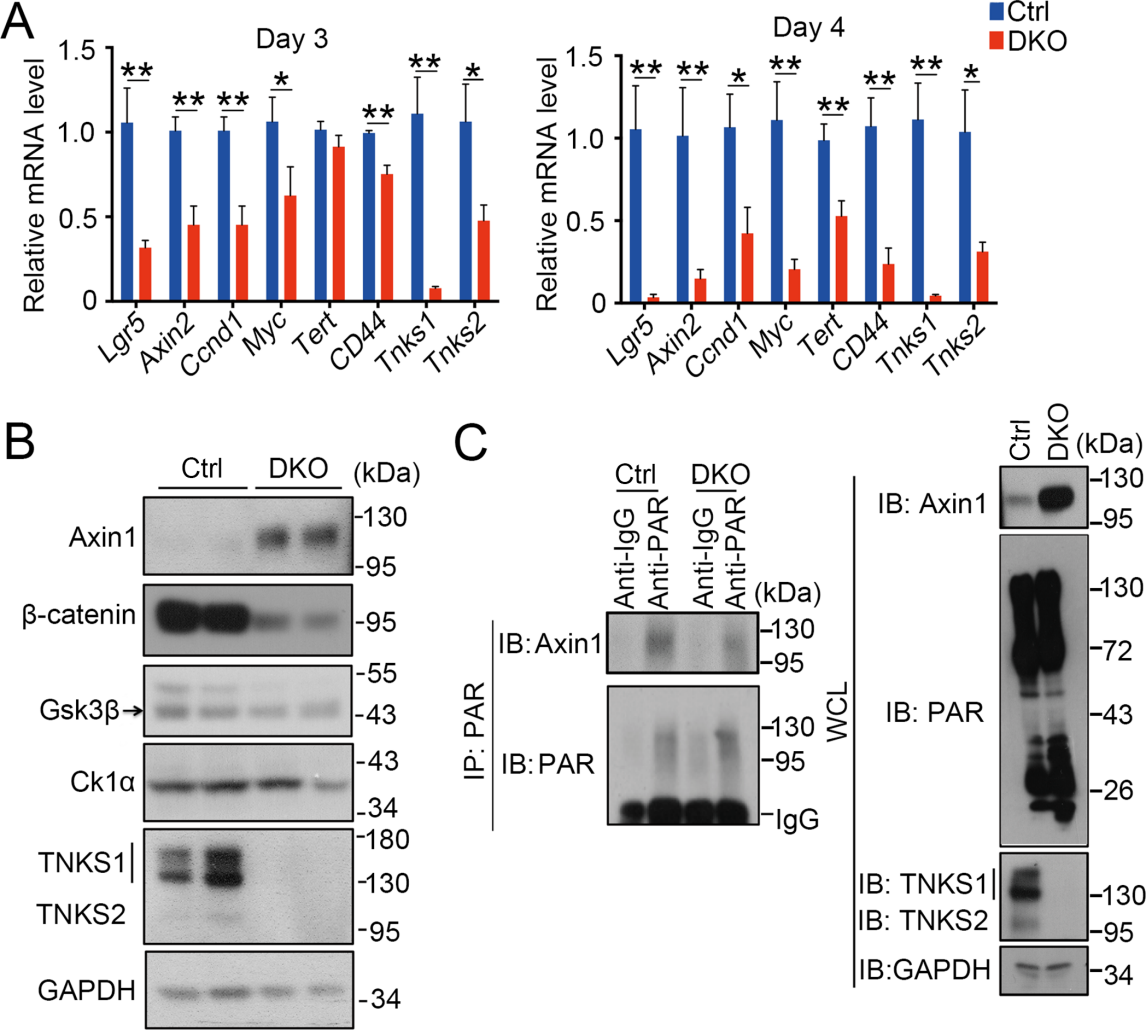
*Tnks* DKO leads to decreased Wnt/β-catenin signaling activity. (A) Crypts were isolated from the mice (n = 3) at day 3 and day 4 after the first TAM injection for qRT-PCR analysis to detect the expression of Wnt targets. Data represent mean ± SD, analyzed by two-way ANOVA test. *P<0.05 and **P<0.01. (B) Immunoblotting analysis of isolated small intestine crypts from control and DKO mice. GAPDH serves as an internal control. The representative result of three independent experiments was shown here. (C) Crypts were isolated from control and DKO mice were at day 4 after first TAM injection for immunoprecipitation (IP) with control IgG or anti-PAR antibody followed by immunoblotting with indicated antibodies. The representative result of three independent experiments was shown here. Protein expression was determined with whole-cell lysates.

Next, we examined Wnt/β-catenin signaling, which is required for the maintenance of proliferative crypt compartment and ISCs in intestine [5]. As Wnt signaling has been indicated to regulate apoptosis [42–44], the observed apoptosis increase in crypts may be caused by deficient Wnt signaling. Indeed, the expression of Wnt target genes started to be decreased at day 2, and dramatically reduced at day 4 (Fig 5A and S5C Fig).

Previous studies reported that TNKSs are positive regulators of Wnt signaling by controlling the stability of Axin [23]. Immunoblotting analysis showed that the protein level of Axin was greatly increased, while total β-catenin was significantly reduced, and GSK3β and CK1α remained the same (Fig 5B). Consistently, parsylation of Axin was reduced in TNKSs DKO crypts (Fig 5C). These data together demonstrate that TNKS deficiency results in Axin stabilization and thus attenuates Wnt signaling.

### Activation of Wnt/β-catenin signaling overcomes TNKSs deficiency-induced growth defect in organoids

**Fig 6:**
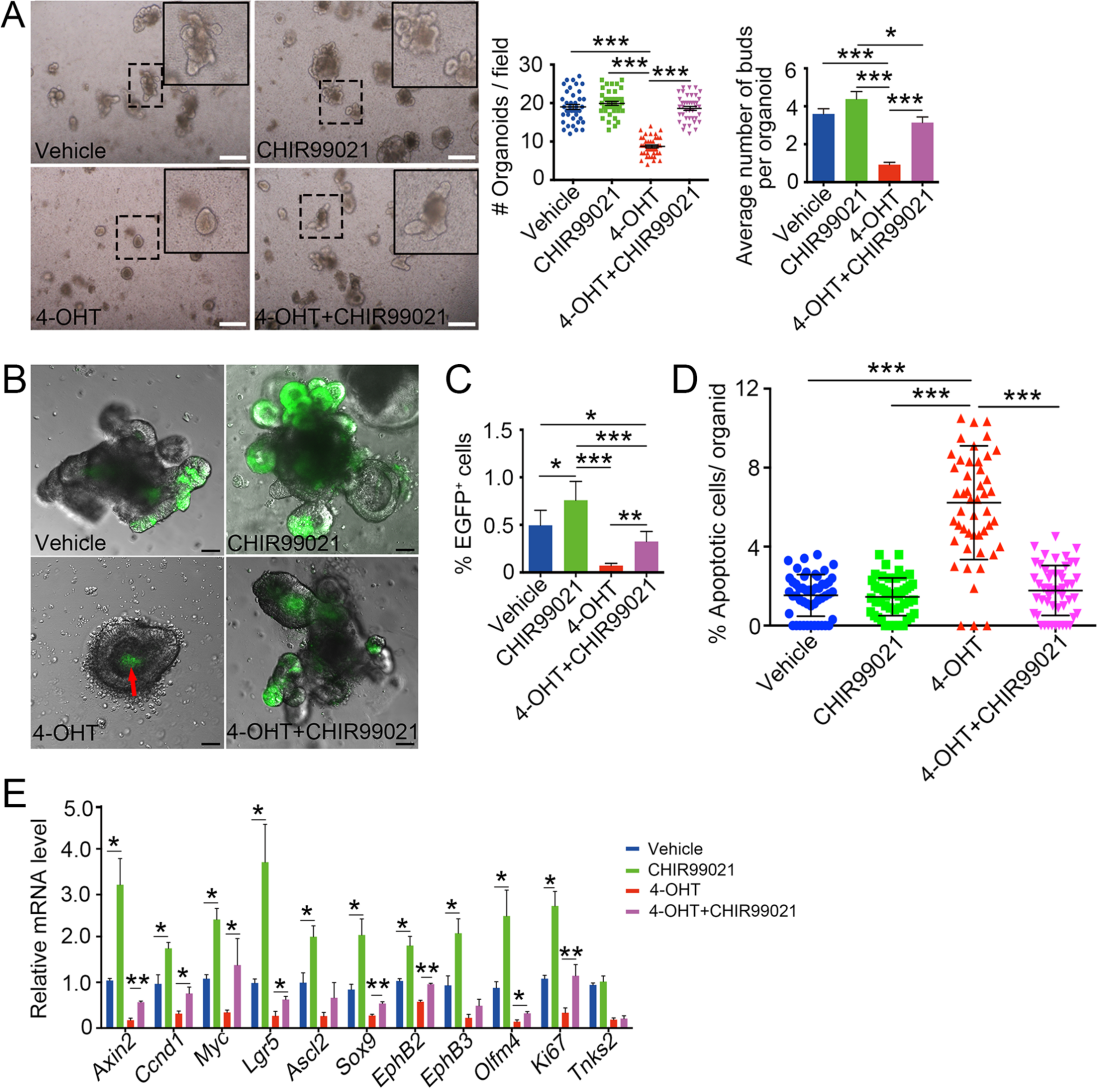
Activation of Wnt/β-catenin signaling by CHIR-99021 restores the growth of *Tnks*-deficient organoids. (A) Small intestine organoid cultures in ENR medium with or without 4-OHT or CHIR-99021. Representative images were taken at 96h, from three independent experiments. Scale bar: 250 μm. Right panel: Quantification of organoids and organoid buds from 40 fields or 50 organoids. (B) Lgr5-EGFP^+^ cell analysis of small intestine organoids in ENR medium with or without 4-OHT or CHIR99021 for 96h from three independent experiments. Scale bar: 50 μm. Red arrow indicates autofluorescence of dead cells in the organoid lumen. (C) Quantification of Lgr5-EGFP^+^ cells from FACS analysis. (D) TUNEL assay of DKO organoids treated with 4-OHT or CHIR99021 for 96h. Apoptotic cells per organoid were accounted from 50 organoids of each condition from 3 independent experiments. (E) DKO organoids cultured for 72h in ENR medium with 4-OHT or CHIR-99021 were harvested for qRT-PCR analysis of the expression of Wnt target genes and stem cell markers. Data from three independent experiments are represented as mean ± SEM, analyzed by unpaired Student’s t-test. *P<0.05, **P<0.01 and ***P<0.001.

To confirm that TNKSs regulate intestinal epithelium renewal mainly by modulating Wnt/β-catenin signaling, we employed the crypt organoid culture system to determine whether the Wnt signaling agonist GSK3β inhibitor CHIR-99021 could overcome *Tnks* deficiency. As shown in Fig 6A, CHIR-99021 restored the organoid survival and the bud formation in the 4-OHT-induced *Tnks* KO organoids. We further observed that CHIR99021 could partially recover Lgr*5*^+^ ISCs in the *Tnks*-deficient organoids (Fig 6B and 6C). We also examined the changes of apoptotic cells and proliferative cells in organoids under these culture conditions. As expected, apoptotic cells were obviously increased in *Tnks* DKO organoids, which were reversed by CHIR-99021 (Fig 6D). In line with it, the expression of ISC markers and Wnt target genes in *Tnks* DKO organoids was partially restored by CHIR-99021 (Fig 6E). In addition, we found that CHIR-99021 could counteract the detrimental effect of the TNKS inhibitor XAV939 on cell growth and apoptosis in *Tnks1*^-/-^ organoids (S6A and S6B Fig). Lastly, we determined whether loss of Apc could abolish the negative effect of XAV939 on organoids as *Apc* mutation leads to the hyperactivation of Wnt signaling. We found that *Apc* mutant organoids derived from the polys of *Villin-Cre*;*Apc*^*+/fl*^ mice could grow normally in the presence of XAV939 (S6A Fig). Similarly, XAV939 had no effect on cell proliferation, apoptosis and the expression of ISC markers and Wnt target genes of *Apc* mutant organoids (S6C and S6D Fig). Together, these data further support the note that TNKSs maintain intestinal homeostasis by controlling Wnt/β-catenin signaling.

## Discussion

In this work, we highlight a previously unrecognized essential role for TNKSs in normal homeostatic self-renewal of adult murine intestine. TNKSs have been indicated to take part in many processes, such as controlling zebrafish gut and tail fin regeneration [45], murine kidney development [46] and murine bone mass maintenance [47]. As these studies used TNKS small molecule inhibitors, it is hard to rule out possible non-specific effects of the small molecules. Therefore, genetics approaches were used to explore their physiological functions. Due to functional redundancy, conventional knockout of single *Tnks* gene showed mild phenotypes [29, 32, 33], while conventional *Tnks* double knockout (*Tnks1*^-/-^;*Tnks2*^-/-^) resulted in embryonic lethality [19]. Thus, we still know little about their *in vivo* roles, especially in tissue homeostasis of adult mammals. In this study, to address the function of TNKSs in intestine, we generated the mice harboring conventional *Tnks1* KO and inducible *Tnks2* conditional KO (*Villin-creERT2*;*Tnks1*^-/-^;*Tnks2*^fl/fl^), and provided strong evidence that both TNKS1 and TNKS2 are essential for the homeostatic maintenance of intestinal epithelium in mice.

Tamoxifen-inducible loss of TNKSs caused a disorganized villi and disrupted crypt structure, leading to mouse death with shorter small intestines at day 10 after the TAM injection. At days 4–5, cell proliferation was reduced, while apoptosis significantly elevated along with a drastically decrease of ISCs in *Tnks* DKO mice, even at day 2 and day 3 after the first tamoxifen administration. This is consistent with a plethora of studies showing that TNKS inhibitors suppress cell proliferation in several cancer cell lines [48–52]. Although inhibition of TNKS activity has been shown to block *Apc* mutant colorectal cancer development [53, 54], we found that the organoids derived from the polys of *Villin-Cre*;*Apc*^*+/fl*^ mice showed normal cell proliferation and activation of Wnt/β-catenin signaling in the presence of XAV939. The discrepancy could be due to the following possibilities: Firstly, *Apc* mutant organoids are cultured in medium containing EGF, Noggin and R-spondin1, which can augment Wnt signaling to support cell growth, while the cells were cultured in a medium without these growth factors. Secondly, R-spondin in organoid culture may have other functions in addition to augment Wnt/β-catenin signaling [55]. Thirdly, the cancer cells may carry other gene mutations. Lastly, the organoids possess three-dimension structure and more than one cell types, while 2D cultured cancer cells only exist one cell type. Interestingly, removal of the only *Tnks* gene in Drosophila shows no obvious phenotypes under normal feeding condition, but ISCs are increased, especially in the posterior midgut [34]. The reduced Wnt signaling due to *Tnks* inactivation in enterocytes may lead to enhanced JAK/STAT signaling in neighboring ISCs non-autonomously and thus their hyperproliferation. Therefore, TNKSs have distinct roles in maintenance of intestinal homeostasis in invertebrates and vertebrates. We also observed that some Paneth cells are dislocated in the villi, and the number of goblet cells decreases in day 5 *Tnks* DKO mice.

As a poly(ADP-ribose) synthase, TNKSs mediate parsylation on various proteins and thus regulate telomere length, mitosis and other cellular processes [20–22]. The degenerative phenotype observed in *Tnks* DKO intestine was associated with increased apoptosis and decreased cell proliferation. Interestingly, the degenerative phenotype was more obvious in small intestine than in colon of DKO mice, which is in agreement with the early reports [56, 57]. It is likely to be caused by the difference in the turnover rate of colonic and small intestinal epithelia, and the pathology of the colon was not dramatic at the time of the death of *Tnks* DKO mice (10 d). By detailed analyses of the underlying mechanism, we found that Wnt signaling activity was greatly reduced due to accumulated Axin protein. Consistent with this, the GSK3β inhibitor CHIR-99021 could maintain the growth of *Tnks* DKO organoids with the recovery of ISCs. The essential role of TNKSs in the maintenance of intestinal epithelium homeostasis is in agreement of the function of Wnt signaling in promoting cell proliferation and inhibiting apoptosis [17]. The results from previous studies showed the high effectiveness of TNKS inhibitors in inhibiting various cancer cell proliferation including CRC [48, 51–53]. However, based on our genetic results and previous pharmacologic results [58], caution should be taken to the possible toxicity of TNKS inhibitors.

## Materials and methods

### Ethics statement

Mice were maintained in the pathogen-free Laboratory Animal Facility of Tsinghua University. The facility has been accredited by AAALAC (Association for Assessment and Accreditation of Laboratory Animal Care International), and the IACUC (Institutional Animal Care and Use Committee) of Tsinghua University approved all animal protocols used in this study by conferring the certificates 12-CYG-2 and 16-CYG-1.

### Mice and in vivo studies

*Villin-cre* mice, *Lgr5-EGFP-IRES-creERT2* and *Apc*^fl/fl^ mice were obtained from Jackson Laboratory. *Tnks1*^+/-^;*Tnks2*^+/fl^ mice have been described previously [19]. *Villin-creERT2* mice were kindly provided by Dr. Sylvie Robine. Both male and female C57BL/6 mice aged 8–10 weeks were used. In general, 2–10 mice per genotype were used in different experiments. In *Tnks2* deletion experiments, mice with the indicated genotype were intraperitoneally injected with 80 mg/kg tamoxifen in sunflower oil at 20 mg/ml for 4–5 consecutive days. Animals were then euthanized at different time points, and tissue was processed immediately. All animal experiments were conducted in accordance with the relevant animal regulations with approval of the Institutional Animal Care and Use Committee of Tsinghua University.

### Antibodies and reagents

XAV939 was purchased from Stemgent. Matrigel was from BD Biosciences. Recombinant mouse Noggin and recombinant human R-Spondin1 were from R&D systems. Recombinant mouse EGF, Advanced DMEM/F12, TrypLE, GlutaMAX, Penicillin/Streptomycin, N2 and B27 were from Invitrogen. N-acetylcysteine, BSA, DAPI, EDTA and Propidium Iodide were from Sigma. Anti-Axin1 antibody was from Cell Signaling Technology (2087s), anti-PAR antibody from Tulip, rabbit anti-Ki67 antibody from Abcam (ab15580), HRP-conjugated anti-mouse IgG and anti-rabbit IgG from GE Healthcare, and other antibodies from Santa Cruz Biotechnology.

### Crypt isolation and organoid culture

Small intestinal crypts were isolated and cultured as previously described [59]. In XAV939 treatment experiments, organoids were cultured in the medium containing EGF, Noggin and R-spondin1 (ENR) for 24h followed by passaging, then cultured in ENR medium with DMSO or XAV939 (50μM) for different time spans. To achieve in vitro *Tnks2* knockout, organoids were cultured in ENR medium for 24h followed by passaging, then incubated in ENR medium with EtOH or 4-OH-tamoxifen (4-OHT, 1μM) for the indicated time. For organoid passaging, cultured organoids were vigorously suspended in cold PBS after removing culture medium and were collected by centrifugation (400g, 3min, 4°C). Then the pelleted organoids were mixed fully with fresh Matrigel, seeded on plate. After Matrigel polymerization, crypt culture medium was added.

### Flow cytometry

To obtain single cell suspension, fresh intestinal crypts isolated from *Lgr5-EGFP-IRES-creERT2* and *Lgr5-EGFP-IRES-creERT2*;*Tnks1*^-/-^;*Tnks2*^fl/fl^ mice were placed in TrypLE (Invitrogen) for 20 min at 37°C. Then the dissociated cells were filtered through a 40μm cell strainer. The EGFP positive cells were subjected to the FACS analysis (MoFlo XDP, Beckman). The acquired data were analyzed by using Summit software.

### mRNA isolation and quantification

RNA was isolated from fresh crypts or cultured organoids by using TRIzol Reagent (Life Technologies). Total RNA yield was determined by using NanoDrop 2000 (Thermo Fisher Scientific). cDNAs were generated using Revertra Ace (Toyobo). Quantitative real-time PCR (qRT-PCR) were carried out in triplicates on the LightCycler 480 (Roche). Primers used were listed in S1 Table.

### TUNEL assay

Using *in situ* cell death detection kit (Roche), apoptosis was assessed by TdT-mediated dUTP nick end labelling (TUNEL) assay in intestinal paraffin section (5μm) according to the manufacturer’s recommendations.

### Immunofluorescence and immunohistochemistry

For immunofluorescence, intestine and organoids were fixed for overnight with 4% formaldehyde at room temperature. Then paraffin intestine or organoid sections were de-paraffinized in isopropanol and dehydrated by a graded alcohol series, followed by antigen retrieval. Next, the section was permeabilized for 10 min in PBS with 0.1% Triton X-100 at room temperature. Then the sections were blocked in 5% BSA/0.1% Triton-X/PBS (blocking buffer) for 1 h at room temperature followed by incubating with the primary antibody at 4°C overnight. The fluorescein-labeled secondary antibodies (1:300, Life Technologies) were added for 1 h at room temperature. Samples were visualized by an Olympus FV1200 Laser Scanning Microscope.

For immunohistochemistry, the paraffin section was prepared as immunofluorescence. Then endogenous peroxidase was quenched by H_2_O_2_. Next, the sections were blocked in blocking buffer for 30 min and incubated overnight with primary antibody at 4°C. Horseradish peroxidase-conjugated secondary antibody (Invitrogen, 1:200) was added for 2 h at room temperature followed by developing with substrate DAB. Then sections were counterstained with hematoxylin and eosin, dehydrated and mounted with resinene.

### Immunoblotting and immunoprecipitation

Protein samples were prepared from fresh intestinal crypts and villi. These two assays were carried out as previously described [59].

### *In situ* hybridization

For *in situ* hybridization experiments, the paraffin sections were de-waxed, rehydrated and hybridized with digoxigenin-labeled probe at 65°C for overnight. After several rounds of wash, sections were placed in blocking solution for 2 h followed by incubating with alkaline phosphatase-conjugated anti-digoxigenin antibody (1:2000; Roche) at 4°C overnight. Then the sections incubated with AP substrate (Roche) after washing several times. *Olfm4* probe (2–1622) was generated with in vitro transcription kit (Roche).

### Statistical analysis

All experiments were performed at least three biological replicates. Data are expressed as mean±SEM or SD, and the statistic significance determined by nonparametric Student’s t-test (Mann–Whitney test) or Two-way ANOVA with GraphPad Prism 6 software. The p value < 0.05 was considered significant and the differences were annotated with asterisks: *(p < 0.05), **(p < 0.01), ***(p < 0.001). The numerical data for statistical analysis in each figure are in S2 Table.

## Supporting information

S1 Fig Deletion of TNKSs results in degeneration of the intestine. (A) Immunohistological analysis of TNKS1 and TNKS2 expression in small intestine of WT mice. Representative images from five mice of each genotype are depicted. Scale bar: 100 μm. (B) Immunohistological analysis of TNKS1/2 knockout efficiency in colon of the mice at day 4 after the first TAM injection. Scale bar: 100 μm. (C) Immunoblotting analysis of TNKS expression in small intestinal crypts of WT, *Tnks1*^-/-^ (*Tnks1*^-/-^;*Tnks2*^fl/fl^) and DKO (*Vil-creERT2*;*Tnks1*^-/-^;*Tnks2*^fl/fl^) mice at day 4 after the first TAM injection. (D) The body weight changes of control and DKO mice (n = 24 each group), administered TAM intraperitoneally daily for 5 times. (E) The faece from mice at day 10 after first TAM injection. (F) The whole gastrointestinal tract from indicated mice at day 10 after first TAM injection. Representative images from 5 mice of each genotype are depicted. Co: colon; SI: small intestine. (G, H) H&E staining of small intestine (G) or colon (H) sections from mice at the indicated times after first TAM injection. Representative images from over 4 mice of each genotype are depicted. Scale bar: 100 μm. Right panel: Quantification of viable crypts of the indicated adult mice (n = 4). Data are represented as means ± SD, analyzed by two-way ANOVA test. *P < 0.05, **P < 0.01, ***P < 0.001.
(TIF)

S2 Fig Loss of TNKSs causes the reduction of proliferative cells. (A-C) Ki67 immunofluorescence analysis of small intestine from mice at day 2 (A), day 3 (B) or day 5 (C) after the first TAM injection. Quantification of Ki67-positive cells was shown from four mice of each genotype. (D) Ki67 immunofluorescence analysis of colon from mice at day 5 after the first TAM injection. Cell nuclei were counterstained with DAPI. Scale bar: 50 μm (A and B) and 80 μm (C and D). Data are represented as means ± SD, analyzed by two-way ANOVA test. *P < 0.05, **P < 0.01, ***P < 0.001.
(TIF)

S3 Fig TNKS DKO enhances apoptosis. TUNEL assay of small intestine of mice at day 2 (A), day 3 (B) or day 5 (C) and colon of mice at day 5 (D) after the first TAM injection. Representative images from 4 mice of each genotype are depicted. Cell nuclei were counterstained with DAPI. Scale bar: 50 μm. Quantification of apoptotic cells in 40 fields for small intestine (n = 4 mice of each genotype) and 30 fields for colon (n = 3 mice of each genotype) was scored. Data are represented as means ± SD, analyzed by two-way ANOVA test. *P < 0.05, **P < 0.01, ***P < 0.001.
(TIF)

S4 Fig Loss of TNKSs reduces Lgr5^+^ stem cells. (A and B) *In situ* hybridization analysis of intestinal stem cell marker *Olfm4* expression in small intestine of control and DKO mice at day 2 and 3 after the first TAM injection. Scale bar: 50 μm. Right panel: Quantification of *Olfm4* positive crypts of the indicated adult mice (n = 4 mice) from 40 fields scored for each genotype mice. Data represent mean ± SD, analyzed by two-way ANOVA test. *P<0.05, **P<0.01 and ***P<0.001. (C) Crypts of indicated mice (n = 3) at day 2 and 3 after the first TAM injection were isolated for analysis of intestinal stem cell marker gene expression by qRT-PCR. Data from three independent experiments are represented as mean ± SEM, analyzed by unpaired Student’s t-test. *P<0.05, **P<0.01 and ***P<0.001.
(TIF)

S5 Fig *Tnks* DKO has no effect on mitosis of intestinal epithelial cells. (A) The percentage of bipolar spindle in crypt cells of the indicated adult mice (n = 5) at day 4 after first TAM injection. 100 mitotic cells were scored for each condition. Data are represented as mean ± SD, analyzed by two-way ANOVA test. Scale bar: 20 μm. (B) Immunofluorescence analysis of Lamin B in crypt cells of mice at day 4 and 5 after the first TAM injection. Representative images from 4 mice of each genotype are depicted. Scale bar: 30 μm. (C) Crypts of mice (n = 3) at day 2 after the first TAM injection were isolated for analysis of Wnt signaling targets by qRT-PCR. Data represent mean ± SD, analyzed by two-way ANOVA test. *P<0.05, **P<0.01 and ***P<0.001.
(TIF)

S6 Fig Inhibition of TNKS activity has no effect on *Apc* mutant organoids. (A) *Tnks1*^-/-^ or *Apc* mutant organoids derived from small intestinal tumors in *Villin-cre;Apc*^+/fl^ mice were cultured in ENR medium with vehicle or XAV939. Representative images were taken at 96h, from three independent experiments. Scale bar: 250 μm. Right panel: Quantification of organoid number. (B) TUNEL assay of *Tnks1*^-/-^ organoids cultured for 96h in ENR medium with CHIR99021 or XAV939. Quantification of apoptotic cells from 50 organoids of each indicated treatment. (C) TUNEL assay and Ki67 immunofluorescence staining analysis from *Apc* mutant organoids cultured in ENR medium with vehicle or XAV939. Representative images were taken at 96h, from three independent experiments. Scale bar = 25 μm. Quantification of Ki67^+^ cells in 50 *Apc* organoids under the indicated treatment. Data (A, B and C) represent mean ± SD, analyzed by two-way ANOVA test. *P<0.05, **P<0.01 and ***P<0.001. (D) *Apc* mutant organoids cultured for 72h in ENR medium with vehicle or XAV939 were harvested for analyzing the expression of Wnt target genes and intestinal stem cell markers by qRT-PCR. Data from three independent experiments are represented as mean ± SEM, analyzed by unpaired Student’s t-test.
(TIF)

S1 Table Primers for RT-qPCR. (DOCX)

S2 Table Numerical data. (XLSX)
