# Supplementary figures and images for: Tankyrases maintain homeostasis of intestinal epithelium by preventing cell death
Source: PLoS Genet. 2018 Sep 27;14(9):e1007697. doi: 10.1371/journal.pgen.1007697 (PMC6177203; doi:10.1371/journal.pgen.1007697)

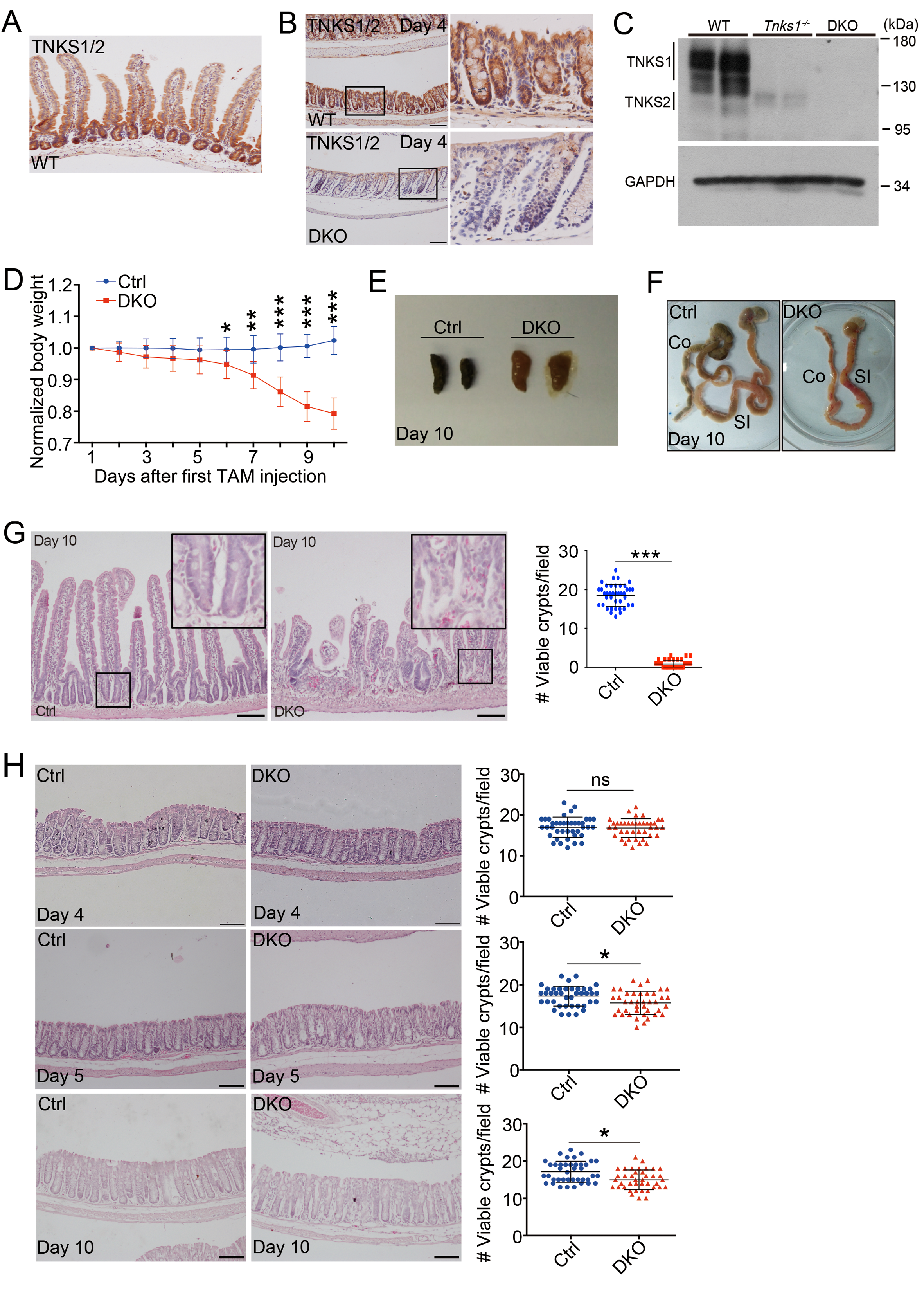

Supplement: S1 Fig — (A) Immunohistological analysis of TNKS1 and TNKS2 expression in small intestine of WT mice. Representative images from five mice of each genotype are depicted. Scale bar: 100 μm. (B) Immunohistological analysis of TNKS1/2 knockout efficiency in colon of the mice at day 4 after the first TAM injection. Scale bar: 100 μm. (C) Immunoblotting analysis of TNKS expression in small intestinal crypts of WT, Tnks1-/- (Tnks1-/-;Tnks2fl/fl) and DKO (Vil-creERT2;Tnks1-/-;Tnks2fl/fl) mice at day 4 after the first TAM injection. (D) The body weight changes of control and DKO mice (n = 24 each group), administered TAM intraperitoneally daily for 5 times. (E) The faece from mice at day 10 after first TAM injection. (F) The whole gastrointestinal tract from indicated mice at day 10 after first TAM injection. Representative images from 5 mice of each genotype are depicted. Co: colon; SI: small intestine. (G, H) H&E staining of small intestine (G) or colon (H) sections from mice at the indicated times after first TAM injection. Representative images from over 4 mice of each genotype are depicted. Scale bar: 100 μm. Right panel: Quantification of viable crypts of the indicated adult mice (n = 4). Data are represented as means ± SD, analyzed by two-way ANOVA test. *P < 0.05, **P < 0.01, ***P < 0.001. (TIF) [file pgen.1007697.s001.tif]

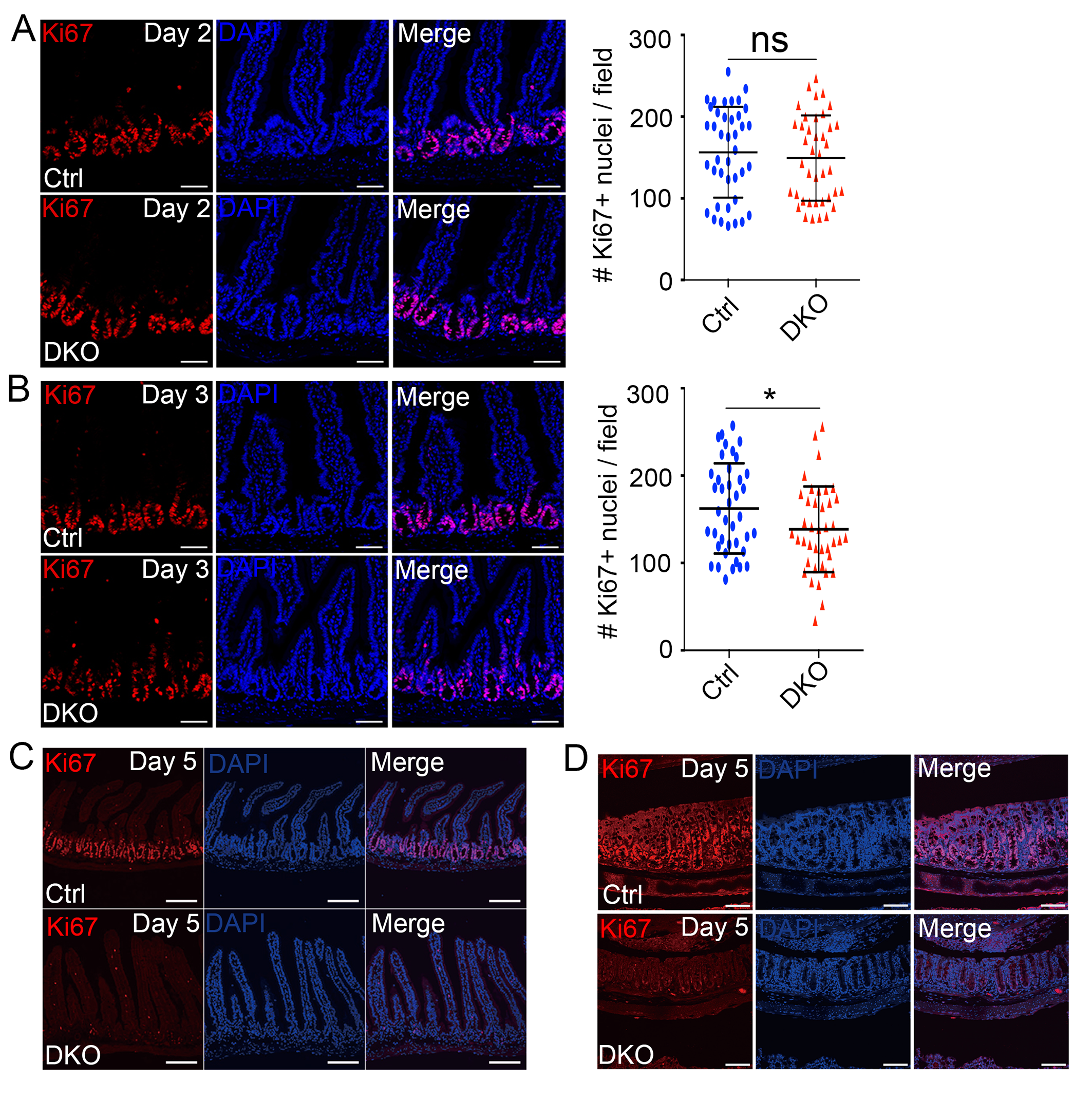

Supplement: S2 Fig — (A-C) Ki67 immunofluorescence analysis of small intestine from mice at day 2 (A), day 3 (B) or day 5 (C) after the first TAM injection. Quantification of Ki67-positive cells was shown from four mice of each genotype. (D) Ki67 immunofluorescence analysis of colon from mice at day 5 after the first TAM injection. Cell nuclei were counterstained with DAPI. Scale bar: 50 μm (A and B) and 80 μm (C and D). Data are represented as means ± SD, analyzed by two-way ANOVA test. *P < 0.05, **P < 0.01, ***P < 0.001. (TIF) [file pgen.1007697.s002.tif]

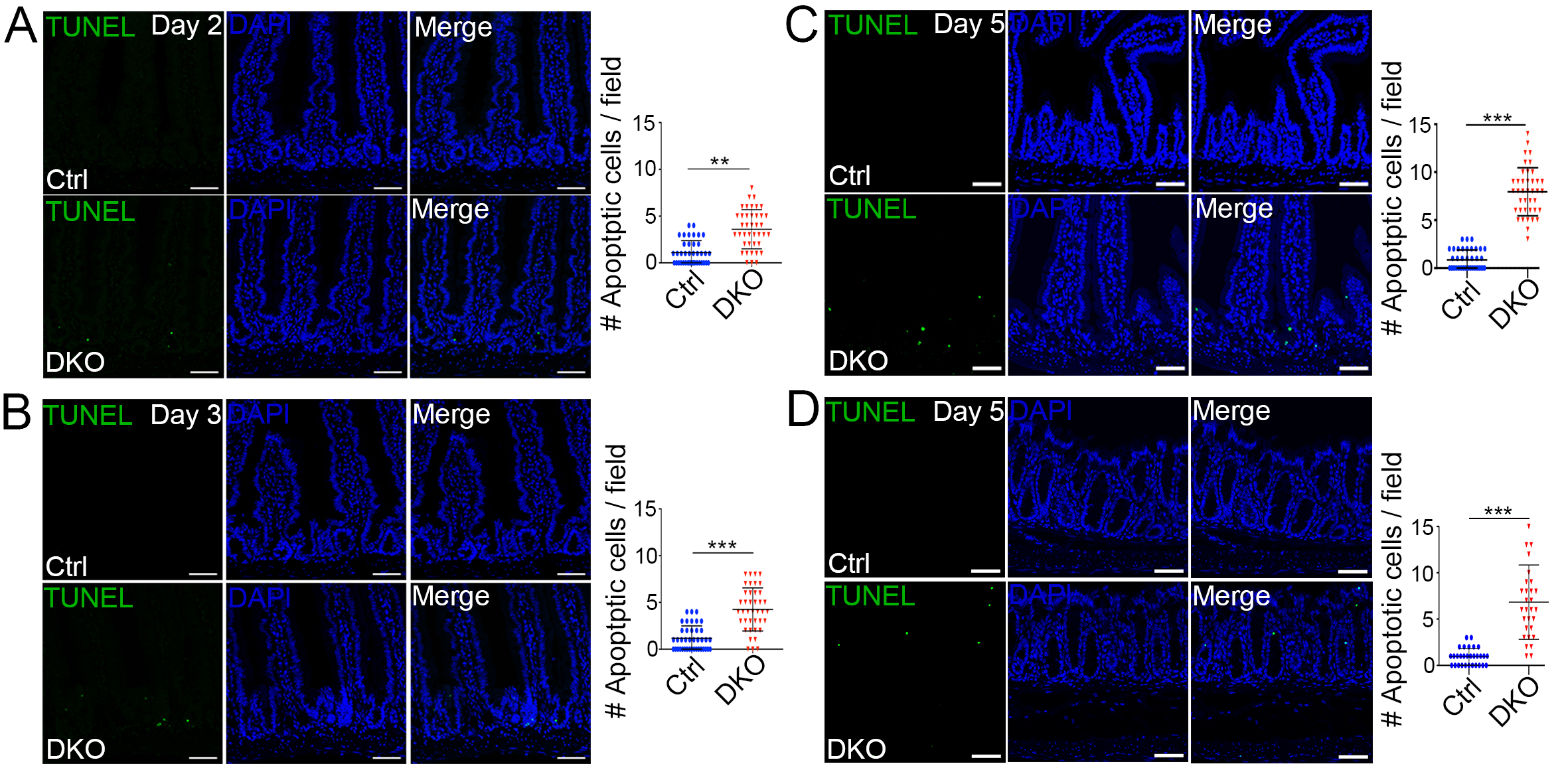

Supplement: S3 Fig — TUNEL assay of small intestine of mice at day 2 (A), day 3 (B) or day 5 (C) and colon of mice at day 5 (D) after the first TAM injection. Representative images from 4 mice of each genotype are depicted. Cell nuclei were counterstained with DAPI. Scale bar: 50 μm. Quantification of apoptotic cells in 40 fields for small intestine (n = 4 mice of each genotype) and 30 fields for colon (n = 3 mice of each genotype) was scored. Data are represented as means ± SD, analyzed by two-way ANOVA test. *P < 0.05, **P < 0.01, ***P < 0.001. (TIF) [file pgen.1007697.s003.tif]

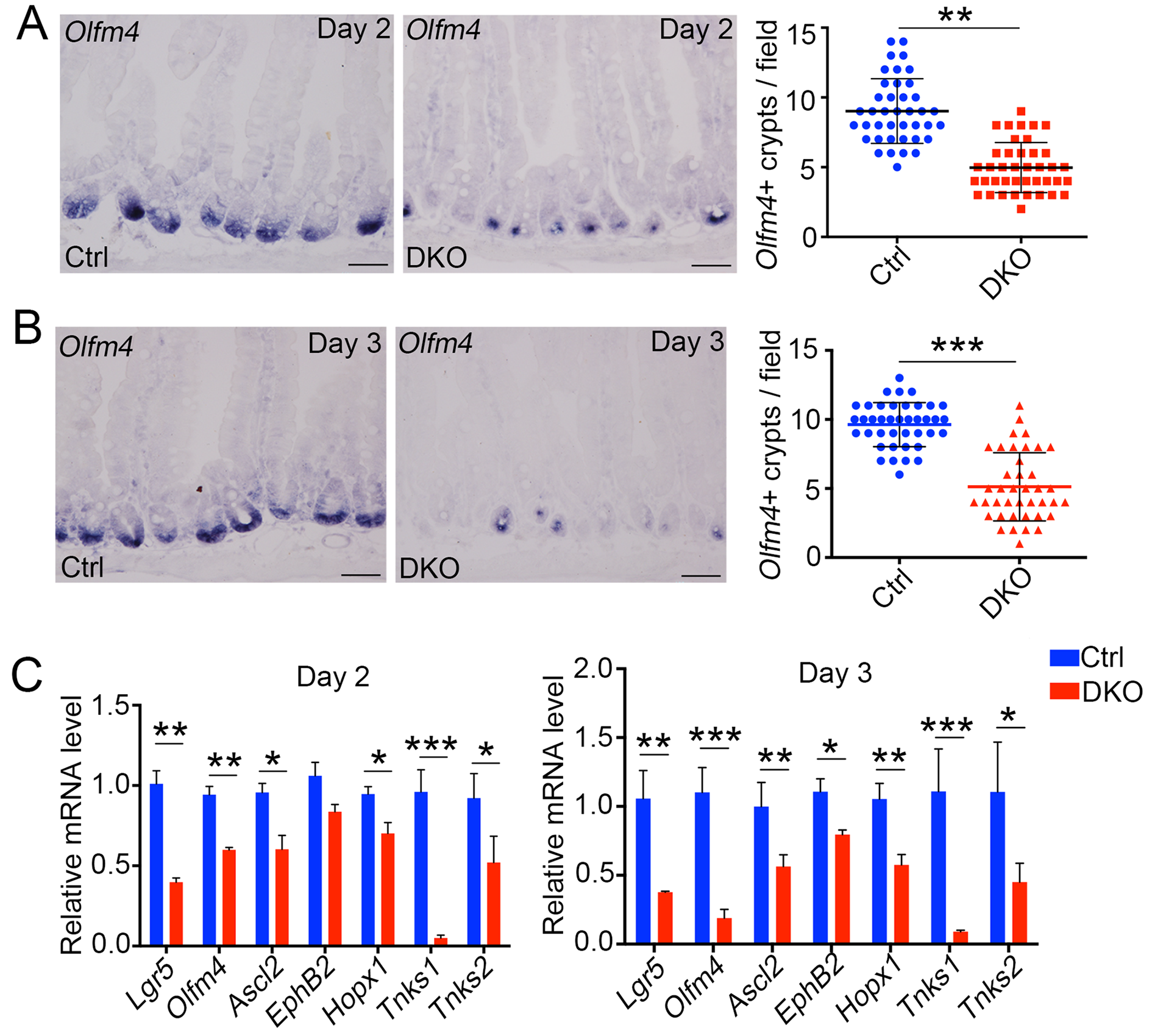

Supplement: S4 Fig — (A and B) In situ hybridization analysis of intestinal stem cell marker Olfm4 expression in small intestine of control and DKO mice at day 2 and 3 after the first TAM injection. Scale bar: 50 μm. Right panel: Quantification of Olfm4 positive crypts of the indicated adult mice (n = 4 mice) from 40 fields scored for each genotype mice. Data represent mean ± SD, analyzed by two-way ANOVA test. *P<0.05, **P<0.01 and ***P<0.001. (C) Crypts of indicated mice (n = 3) at day 2 and 3 after the first TAM injection were isolated for analysis of intestinal stem cell marker gene expression by qRT-PCR. Data from three independent experiments are represented as mean ± SEM, analyzed by unpaired Student’s t-test. *P<0.05, **P<0.01 and ***P<0.001. (TIF) [file pgen.1007697.s004.tif]

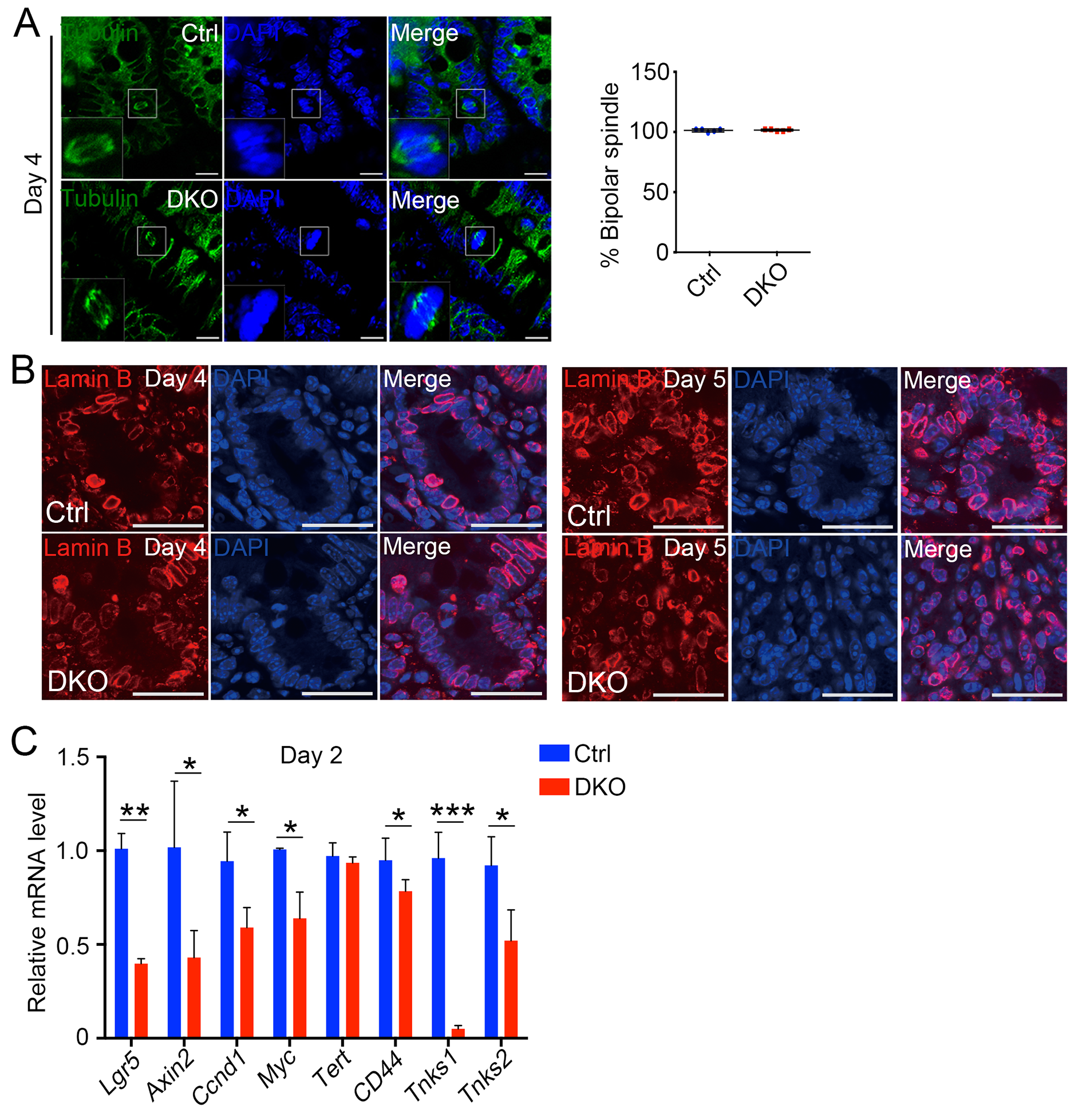

Supplement: S5 Fig — (A) The percentage of bipolar spindle in crypt cells of the indicated adult mice (n = 5) at day 4 after first TAM injection. 100 mitotic cells were scored for each condition. Data are represented as mean ± SD, analyzed by two-way ANOVA test. Scale bar: 20 μm. (B) Immunofluorescence analysis of Lamin B in crypt cells of mice at day 4 and 5 after the first TAM injection. Representative images from 4 mice of each genotype are depicted. Scale bar: 30 μm. (C) Crypts of mice (n = 3) at day 2 after the first TAM injection were isolated for analysis of Wnt signaling targets by qRT-PCR. Data represent mean ± SD, analyzed by two-way ANOVA test. *P<0.05, **P<0.01 and ***P<0.001. (TIF) [file pgen.1007697.s005.tif]

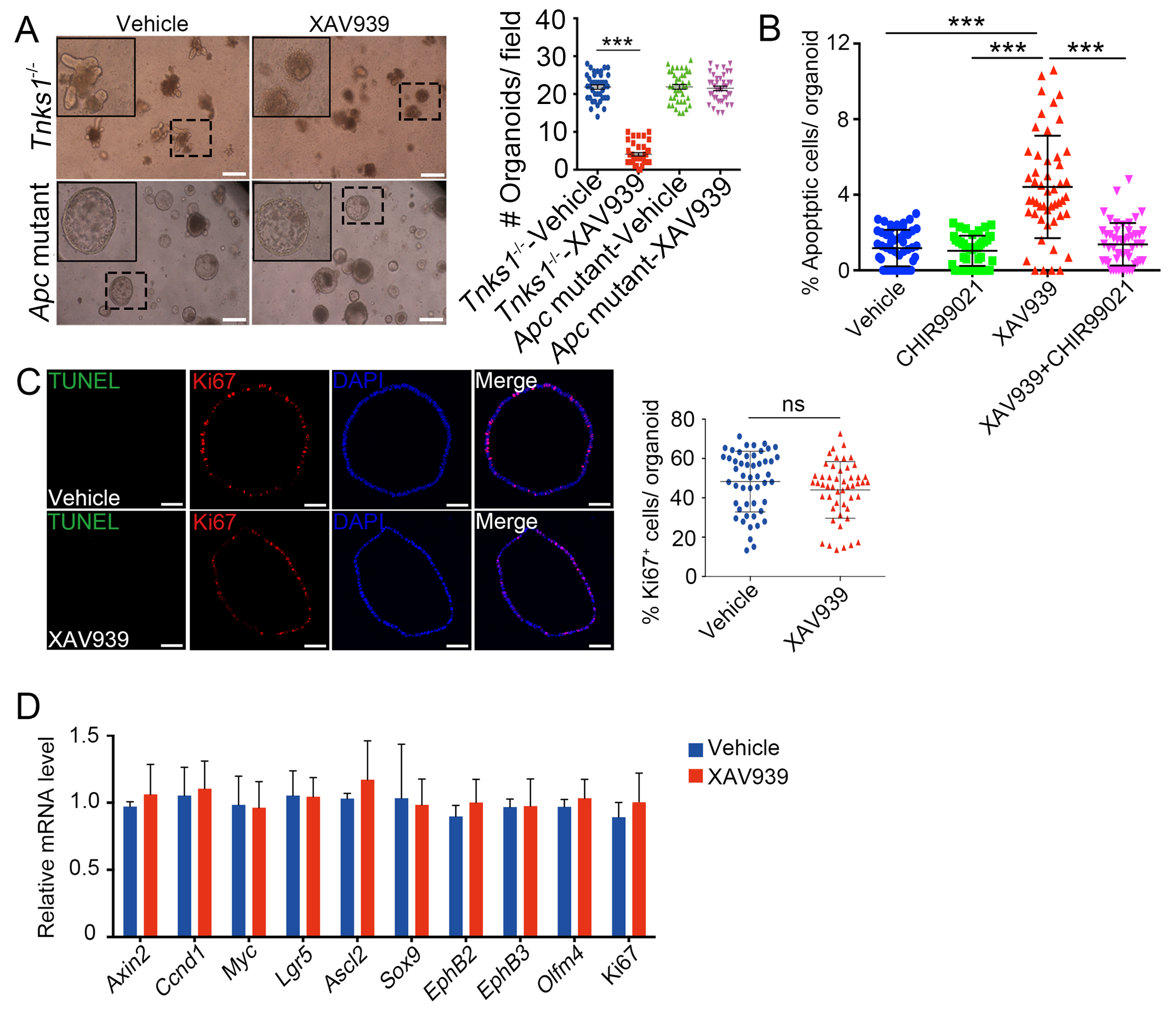

Supplement: S6 Fig — (A) Tnks1-/- or Apc mutant organoids derived from small intestinal tumors in Villin-cre;Apc+/fl mice were cultured in ENR medium with vehicle or XAV939. Representative images were taken at 96h, from three independent experiments. Scale bar: 250 μm. Right panel: Quantification of organoid number. (B) TUNEL assay of Tnks1-/- organoids cultured for 96h in ENR medium with CHIR99021 or XAV939. Quantification of apoptotic cells from 50 organoids of each indicated treatment. (C) TUNEL assay and Ki67 immunofluorescence staining analysis from Apc mutant organoids cultured in ENR medium with vehicle or XAV939. Representative images were taken at 96h, from three independent experiments. Scale bar = 25 μm. Quantification of Ki67+ cells in 50 Apc organoids under the indicated treatment. Data (A, B and C) represent mean ± SD, analyzed by two-way ANOVA test. *P<0.05, **P<0.01 and ***P<0.001. (D) Apc mutant organoids cultured for 72h in ENR medium with vehicle or XAV939 were harvested for analyzing the expression of Wnt target genes and intestinal stem cell markers by qRT-PCR. Data from three independent experiments are represented as mean ± SEM, analyzed by unpaired Student’s t-test. (TIF) [file pgen.1007697.s006.tif]
