# Supplementary material for: Tankyrases maintain homeostasis of intestinal epithelium by preventing cell death
Source: PLoS Genet. 2018 Sep 27;14(9):e1007697. doi: 10.1371/journal.pgen.1007697 (PMC6177203; doi:10.1371/journal.pgen.1007697)
Supplement: S1 Table — (DOCX) [file pgen.1007697.s007.docx]

**S1 Table. Primers for RT-qPCR**

| Gene | Forward | Reverse |
| --- | --- | --- |
| *Gapdh* | AAGAAGGTGGTGAAGCAG | TCATACCAGGAAATGAGC |
| *Lgr5* | CGGGACCTTGAAGATTTCCT | GATTCGGATCAGCCAGCTAC |
| *Ascl2* | GCCTGACCAAATGCCAAGTG | ATTTCCAAGTCCTGATGCTGC |
| *Olfm4* | CGAGACTATCGGATTCGCTATG | TTGTAGGCAGCCAGAGGGAG |
| *Axin2* | GCTCCAGAAGATCACAAAGAGC | AGCTTTGAGCCTTCAGCATC |
| *Myc* | GCTGTTTGAAGGCTGGATTTC | GATGAAATAGGGCTGTACGGAG |
| *Sox9* | GCCAGATGGACCCACCAGTAT | TCCAAACAGGCAGGGAGATTC |
| *Ccnd1* | GCCATCCAAACTGAGGAAAA | AGGTAAGGGCCATCTGAAAACT |
| *Hopx1* | AGGAGCAGACGCAGAAATG | GAAACATCAAAACAGCCTGGG |
| *EphB2* | AGAATGGTGCCATCTTCCAG | GCACATCCACTTCTTCAGCA |
| *EphB3* | CATGGACACGAAATGGGTGAC | GCGGATAGGATTCATGGCTTCA |
| *CD44* | GGCAGAAGAAAAAGCTGGTG | TCTGGGGTCTCTGATGGTTC |
| *Ki67* | CCACACAGATGCCCTGTAAT | CTCTACTTTCCCACGTCTTGTC |
| *Tert* | GTGTGATTCAGCTTCCCTTT | CCTTTAGTGTCATTCCTGGAT |
| *Tnks1* | CCCCGACTTCGTCTTCGTCT | CACGTCCCCATTCCGACAGG |
| *Tnks2* | CGAGCTGGCAGGAGTGGTC | TCCGCTCCCCGCTGTCACCA |
